# Intricate trophic links between threatened vertebrates confined to a small island in the Atlantic Ocean

**DOI:** 10.1002/ece3.5105

**Published:** 2019-04-01

**Authors:** Ricardo J. Lopes, Catarina J. Pinho, Bárbara Santos, Mariana Seguro, Vanessa A. Mata, Bastian Egeter, Raquel Vasconcelos

**Affiliations:** ^1^ CIBIO, Centro de Investigação em Biodiversidade e Recursos Genéticos, InBIO Laboratório Associado Universidade do Porto Vairão Portugal; ^2^ Departamento de Biologia, Faculdade de Ciências Universidade do Porto Porto Portugal

**Keywords:** birds, Cabo Verde, DNA metabarcoding, endemics, reptiles, trophic networks

## Abstract

Trophic networks in small isolated islands are in a fragile balance, and their disturbance can easily contribute toward the extinction vortex of species. Here, we show, in a small Atlantic island (Raso) in the Cabo Verde Archipelago, using DNA metabarcoding, the extent of trophic dependence of the Endangered giant wall gecko *Tarentola gigas* on endemic populations of vertebrates, including one of the rarest bird species of the world, the Critically Endangered Raso lark *Alauda razae*. We found that the Raso lark (27%), Iago sparrow *Passer iagoensis* (12%), Bulwer's petrel *Bulweria bulwerii* (15%), and the Cabo Verde shearwater *Calonectris edwardsii* (10%) are the most frequent vertebrate signatures found in the feces of the giant wall gecko. This work provides the first integrative assessment of their trophic links, an important issue to be considered for the long‐term conservation of these small and isolated island ecosystems.

## 1 INTRODUCTION

Small islands, due to their size, long‐lasting barriers to dispersal and occurrence of small populations adapted to these atypical environments, are particularly exposed to climatic, environmental and anthropogenic pressures that increase the probability of extinction of native populations (Whittaker & Fernández‐Palacios, 2007). Species relationships, especially competition and predation, can also have a large impact on the viability of populations (Holt, 2010), since trophic networks in small islands can be more unstable than in continental grounds or in larger islands (Novosolov, Rodda, Gainsbury, & Meiri, 2018). Indeed, there is a trend toward smaller food networks (Roslin, Varkonyi, Koponen, Vikberg, & Nieminen, 2014) also facilitated by the fact that smaller islands usually hold lower species diversity than larger islands (Whittaker & Fernández‐Palacios, 2007). These factors may lead to a higher probability of collapse of trophic networks due to trophic cascades or stochastic environmental processes, such as drought or hurricanes (Massol et al., 2017). On the other hand, these smaller trophic networks can provide better analytical frameworks to test alternative hypothesis concerning the impact of biogeographical gradients on trophic metrics, due to their simplicity, lower number of confounding variables, and the possible replication of food webs in multiple islands (Gravel, Massol, Canard, Mouillot, & Mouquet, 2011; Matias et al., 2017; Roslin et al., 2014; Spiller & Schoener, 1996). However, while the impact of new invasive vertebrate species on these small food webs (McCreless et al., 2016; Medina et al., 2011; Zarzoso‐Lacoste et al., 2016) or on vertebrate diets based on invertebrates or plants (Kartzinel & Pringle, 2015) has been extensively documented, the analysis of insular food webs with a strong component of vertebrate predation is less frequent. This is a result of the natural lack of secondary vertebrate consumers in many small islands and also from the difficulty of retrieving data concerning these links in these remote, small, and vulnerable communities.

**Figure 1:**
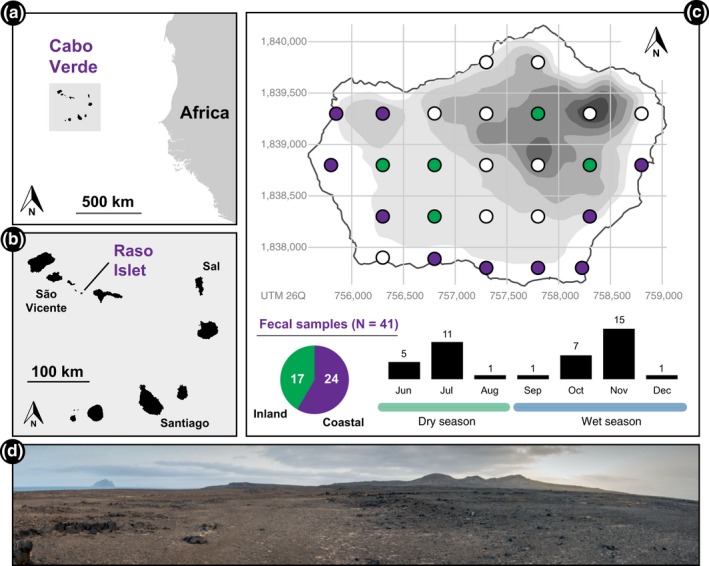
(a) Geographic location of the Cabo Verde Archipelago and (b) Raso Islet; (c) The surveyed areas in Raso, in a 500 m grid. White circles show the areas where no gecko was found or sampled. Colored circles show the areas with positive fecal sampling (violet: coastal; green: inland). Also, shown is the spatial and monthly discrimination of the number of feces analyzed; (d) Panoramic view of the main plateau of Raso and the highest elevations during the dry season

Cabo Verde (Figure 1b) has been recognized as one of the most important areas for conservation within the Mediterranean Basin Biodiversity Hotspot, and is the only tropical member of the Macaronesian Region (Mittermeier, Turner, Larsen, Brooks, & Gascon, 2011; Myers, Mittermeier, Mittermeier, Fonseca, & Kent, 2000). Here, we focus on the small and threatened vertebrate community of Raso, a protected uninhabited islet in this archipelago (Figure 1c). It comprises six colonial seabird species, nine terrestrial breeding bird species, and four species of reptiles (Table 1).

**Table 1:** Vertebrate species known to breed in Raso Islet, according to Vasconcelos, Brito, Carranza, & Harris (2013) and Hazevoet (2015). The symbol "•" represents a species with confirmed breeding records while "?" represents a species suspected to breed or have bred

Group	Common name	Scientific name	Raso
Marine birds	Cabo Verde shearwater	*Calonectris edwardsii *(Oustalet, 1883)	•
	Boyd's shearwater	*Puffinus boydi* *Mathews*, 1912	•
	Bulwer's petrel	*Bulweria bulwerii *(Jardine and Selby, 1828)	•
	Cabo Verde storm petrel	*Oceanodroma jabejabe *(Bocage, 1875)	•
	Red‐billed tropicbird	*Phaethon aethereus *(Linnaeus, 1758)	•
	Brown booby	*Sula leucogaster *(Boddaert, 1783)	•
Terrestrial birds	Little egret	*Egretta garzetta *(Linnaeus, 1866)	?
	Osprey	*Pandion haliaetus *(Linnaeus, 1758)	•
	Neglected kestrel	*Falco neglectus *Schlegel, 1873	•
	Quail	*Coturnix coturnix *(Linnaeus, 1758)	•
	Cream‐colored courser	*Cursorius cursor *(Latham, 1787)	?
	Cabo Verde barn owl	*Tyto detorta *Hartert, 1913	•
	Raso lark	*Alauda razae *(Alexander, 1898)	•
	Brown‐necked raven	*Corvus ruficollis *Lesson, 1831	•
	Iago sparrow	*Passer iagoensis *(Gould, 1837)	•
Reptiles	Bouvier's leaf‐toed gecko	*Hemidactylus bouvieri *(Bocourt, 1870)	•
	Raso wall gecko	*Tarentola raziana *Schleich, 1984	•
	Giant wall gecko	*Tarentola gigas *(Bocage, 1875)	•
	Stanger's skink	*Chioninia stangeri *(Gray, 1845)	•

Of special concern are the populations of the Raso lark *Alauda razae* and the giant wall gecko *Tarentola gigas*, that coexist in this islet since its origin, a couple of million years ago. Considered one of the rarest bird species of the world (Donald, Collar, Marsden, & Pain, 2013), the Raso lark is a Critically Endangered ground‐nesting bird presently restricted to Raso (BirdLife International, 2017; Hirschfeld, Swash, & Still, 2013). With <1,500 individuals, this resident population is subject to substantial size fluctuations, mostly due to the stochasticity of environmental conditions but also due to predation (Brooke, n.d.). In some years, a considerable egg predation was recorded, without a clear identification of the predator(s), although the researchers hypothesize the giant wall gecko *T. gigas* as being the most likely candidate (Donald et al., 2005; Donald, Ponte, Groz, & Taylor, 2003). This large nocturnal gecko, with an average snout‐vent length >10 cm (Vasconcelos, Perera, Geniez, Harris, & Carranza, 2012) presently only occurs on Raso and nearby Branco islets. It is classified as Endangered due to the small population size and restricted range of occupancy (Vasconcelos, 2013).

Previous studies of the diet of this nocturnal gecko, relying on traditional methodologies, have already shown evidence of a generalist diet. Morphological analysis of gecko feces and gut content, effective for identifying diet items with nondigestible parts, recorded the presence of plants, invertebrates, fish scales and seabird, and small bird feathers (Mateo, Geniez, Hernández‐Acosta, & Jurado, 2016; Schleich, 1980). Observations also confirm that this gecko often eats regurgitated food, egg remains, whole eggs and possibly chicks and feces from some of the most abundant seabirds, the Near Threatened Cabo Verde shearwater *Calonectris edwardsii,* and the Least Concern Bulwer's petrel *Bulweria bulwerii* (den Hartog, 1990; Schleich & Wutke, 1983; Schleich, 1980). The importance of passerines as diet items is of special concern, since geckos may feed on eggs and possibly nestlings. The evidence to date lead to the hypothesis that this gecko is the major (and perhaps only) natural predator of eggs of the Raso lark (Donald et al., 2005, 2003), and possibly of the Iago sparrow *Passer iagoensis*, the other abundant resident passerine species.

In 2016, we deployed a spatially and seasonally unbiased sampling of fecal samples of the giant wall gecko using DNA metabarcoding. This technique maximizes resolution, detection of rare events, and detection of soft, small, and invisible prey items, and ultimately can decrease biases of traditional methods (Nielsen, Clare, Hayden, Brett, & Kratina, 2017; Pompanon et al., 2012; Roslin & Majaneva, 2016). We compared the diets of this gecko in Raso and Branco islets during the wet season of 2016 (Pinho et al., 2018). Using 23 samples and multiple primers, it was possible to record in Raso the presence of plants, invertebrates, and vertebrates, including the Raso lark (four samples), Iago sparrow (three samples), Cabo Verde shearwater (two samples), Stanger's skink *Chioninia stangeri* (one samples), and two fishes. In Branco, the most abundant vertebrate was the Cabo Verde shearwater (seven out of 28 samples).

The confirmation of Raso lark in gecko diet is particularly relevant to current conservation actions as translocation of Raso larks to the neighboring Santa Luzia Island commenced in April 2018 (Geraldes, Kelly, Melo, & Donald, 2016) and plans for translocation of giant wall gecko to the same island are currently being evaluated. The decision to undertake the proposed gecko translocation will depend largely on the anticipated impact this might have on the recently translocated Raso lark populations.

The aim of the present study was to further characterize the vertebrate portion of the diet of giant wall geckos, using all samples collected in Raso across both the wet and dry seasons, with the primary goal of clarifying the trophic links, especially between these two species of conservation concern: the giant wall gecko and the Raso lark.

## 2 MATERIALS AND METHODS

### 2.1 Sampling

Raso is located in the uninhabited Santa Luzia Marine Reserve, which comprises one island and two islets that hold important endemic species, facing continuous human pressure (Vasconcelos, Freitas, & Hazevoet, 2015). With a land area around 6 km^2^ (Figure 1c), Raso is characterized by plains and low altitude arid zones with patches of grassy vegetation (see Figure 1d; Freitas, Hazevoet, & Vasconcelos, 2015).

A total of 71 giant wall gecko fecal samples were collected from June to December 2016 to encompass the most critical periods of breeding of the seabird species and the Raso lark (Vasconcelos et al., 2015), using a point transect approach to ensure unbiased spatial sampling (Figure 1c). The island was divided in 500 m quadrats that were surveyed applying a similar effort rate (Doan, 2016). Individual geckos were captured by hand, sexed (through the observation of morphological differences), measured (snout‐vent length), and marked using a subcutaneous RFID implantable transponder (Dorset Identification, The Netherlands) to ensure that each individual was not sampled twice (Ferner & Plummer, 2016). An abdominal massage was performed for the release of fecal pellets, which were preserved in tubes with 96% ethanol and refrigerated at 4°C as soon as possible until processed in the laboratory.

### 2.2 DNA extraction and sequencing

Fecal samples were dried in an incubator at 50°C before DNA extraction and two DNA elutions (50 μl each) were extracted using the Stool DNA Isolation Kit (Norgen Biotek Corporation, Canada) following the manufacturer's protocol.

Since we aimed to quantify vertebrate links, due to their conservation importance, we chose to amplify a V5‐loop fragment of the mitochondrial 12S gene (73–110 base pairs) to correctly identify vertebrate prey types (Table 1). This was performed using the primers 12sv5F (5′ ‐TAGAACAGGCTCCTCTAG ‐3′) and 12sv5R (5′ ‐TTAGATACCCCACTATGC ‐3′) designed by Riaz et al. (2011) and already validated in several studies (De Barba et al., 2014; Kocher et al., 2017; Shehzad et al., 2012). They were then modified to contain Illumina adaptors and a five base pair individual identification barcode. A blocking primer was also designed to prevent amplification of *T. gigas* DNA. For this, we built an alignment using available 12S sequences of this species as well as of birds and fishes known to occur in Cabo Verde or of taxonomically related species and designed the blocking primer to overlap with 12sv5F (*T. gigas* blocking primer: 5′‐ CCCCACTATGCTCAACCGTTAACAAAG‐(C3 spacer) ‐3′), following recommendations by Vestheim and Jarman (2008).

Library preparation followed the MiSeq protocol for 16S Metagenomics (Illumina). PCR reactions were carried out in volumes of 25 µl, comprising 10.4 µl of QIAGEN Multiplex PCR Master Mix (Qiagen), 0.4 µl of each 10 µM primer, 8 µl of 10 µM blocking primer, 2.8 µl of ultra‐pure water, and 3 µl of DNA extract. Cycling conditions used initial denaturing at 95°C for 15 min, followed by 39 cycles of denaturing at 95°C for 30 s, annealing at 48°C for 30 s and extension at 72°C for 30 s, with a final extension at 72°C for 10 min. Amplification success was checked by visually inspecting 2 μl of each PCR product on a 2% agarose gel. PCR products were purified using Agencourt AMPure XP beads (Beckman Coulter) and subsequently quantified using Nanodrop (Thermo Scientific) and diluted to similar concentrations. Samples amplified with different barcodes were pooled together and Illumina indexes were added to the pooled PCR products using the Nextera XT Kit (Illumina), allowing individual identification of each amplified product. PCR reactions and cycling conditions were similar to the ones of the first PCR except that only eight cycles of denaturing, annealing and extension were done, with annealing at 50°C. PCR products were again purified, quantified and pooled at equimolar concentrations (15 nM). The final library was quantified using qPCR with a KAPA Library Quant Kit qPCR Mix (KAPA Biosystems), on the iCycler Real‐Time PCR Detection System (Bio‐Rad), diluted to 4 nM, and run in a MiSeq sequencer (Illumina) using a 2 × 250 bp MiSeq Reagent Kit (Illumina) for an expected average of 12,000 paired‐end reads per sample.

### 2.3 Bioinformatics and data analysis

Bioinformatic processing of sequencing reads was done using OBITools (Boyer et al., 2016). Paired‐end reads were aligned (command *illuminapairedend*) and discarded if alignment score was <40. Reads were then assigned to samples and primer sequences were removed (command *ngsfilter*), allowing a total of four mismatches to the expected primer sequence. Finally, reads were collapsed into haplotypes and singletons (haplotypes with only one read) were removed. Potentially spurious sequences with an “r” level of one were removed (command *obiclean*), meaning that any “A” haplotype differing one base pair from a “B” haplotype, with an absolute read count lower than “B,” and that was not found without the presence of “B” in any sample, was removed (assumed to be most likely a PCR or sequencing error). The PCRs with <100 reads in total after this step were considered to have failed and removed. For the remaining ones, any haplotype representing <1% of the reads obtained for that PCR was also removed (Mata et al., 2016).

Prey items were identified by comparing the final set of haplotypes against the online GenBank database (Benson et al., 2013), as well as unpublished sequences of vertebrates collected on Raso. Sequences with <90% similarity between known species were only classified to the class level, while those with similarity between 90% and 95% were classified to the family level. Sequences with more than 95% of similarity between known species were classified to the species or genus level. When the same haplotype matched more than one species or genus with similar probabilities, we only considered species or genera known to occur on Raso Islet, or on other islands in Cabo Verde. After identifying all the haplotypes, we removed haplotypes from several vertebrates (e.g., human and pig), due to the high probability of being lab contaminations.

After these processing steps, a total of 41 fecal samples remained and the frequency of occurrence of each prey item in the overall fecal sample size and the respective 95% confidence interval (95% CI) were calculated in R 3.4.1 using the *binom.test* command (R Core Team, 2017) (Supporting Information Table S1 in Dryad repository).

## 3 RESULTS

**Figure 2:**
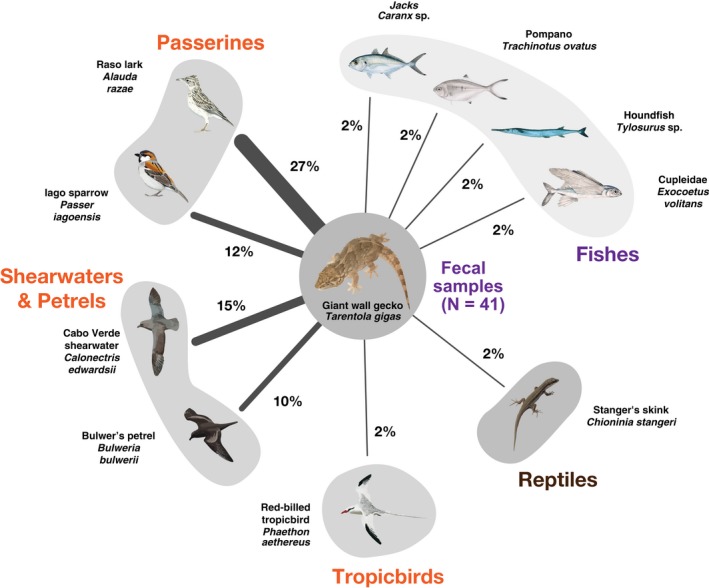
Vertebrate species observed in the diet of the giant wall gecko are shown in a network where links width is positively correlated to the frequencies of occurrence of each vertebrate in the diet. Values shown near each link as percentages frequency of occurrence of a sample size of 41

A total of 33 vertebrate signatures occurred in 22 (54%) of the feces (average of 0.8 prey items per feces). The most frequent items in the feces were passerines (Raso lark and Iago sparrow) and seabirds (Bulwer's petrel and the Cabo Verde shearwater), while one tropicbird, one pelecaniform bird, one reptile, and four fishes occurred only once (Figure 2 and Supporting Information Table S1 in Dryad repository).

Raso lark was present in 27% (95% CI = 0.14–0.43) of the samples, while the Iago sparrow was present in 12% (95% CI = 0.004–0.26). Bulwer's petrel was present in 15% (95% CI = 0.05–0.29) and Cabo Verde shearwater present in 10% (95% CI = 0.03–0.23). All other items were only found once (each in 2% of the samples, 95% CI = 0.00–0.13), including the red‐billed tropicbird *Phaethon aethereus*, Stanger's skink *C. stangeri,* and several fishes, such as jacks *Caranx* sp., blue flying fish *Exocoetus volitans*, pompano *Trachinotus ovatus,* and needlefishes *Tylosurus* sp.. One bird species, also found once, was only possible to assign to the Pelecaniformes order. Since the Sulidae family is still considered by GenBank as belonging to this order, the most likely assignment would be the breeding species brown booby *Sula leucogaster*.

**Figure 3:**
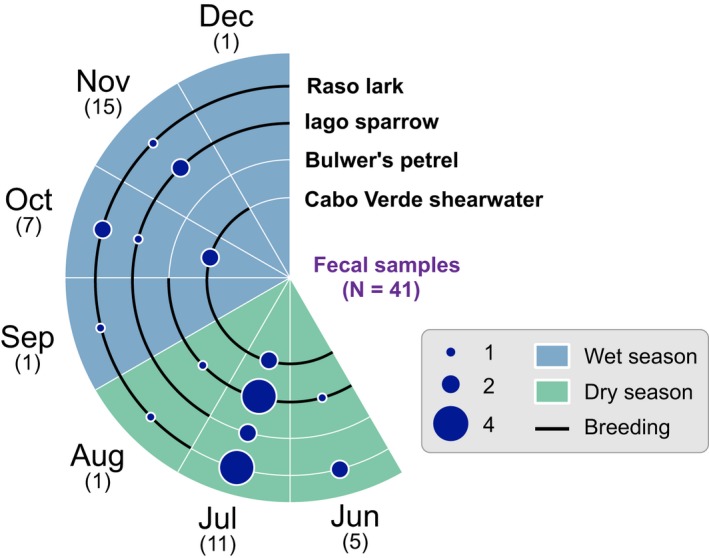
Monthly variation of the four main vertebrate species recorded in the diet of the giant wall gecko. Each radial plot corresponds to one of these species. The circle sizes are positively correlated with the number of positive records in the diet. For each bird species, we also show the duration of their breeding time (black line) and months is colored according to the season (dry or wet). Sample size for each month is shown in brackets, below each month label

Concerning the four species with higher incidence in the feces, the Raso lark was mainly present in samples collected in July and October, while the Iago sparrow was mostly observed in samples from July and November. In the case of the seabirds, Bulwer's petrel occurred mainly in July, while the Cabo Verde shearwater occurred mainly in July and October (Figure 3).

## 4 DISCUSSION

Our results show that giant wall gecko diet is linked to most of the available and more abundant terrestrial vertebrates, including one of the rarest Critically Endangered ground‐nesting bird in the world, the Raso lark. This is a viable strategy that can occur in large ectotherms to increase their diet breadth in small and resource‐limited areas, characterized by low species diversity (Pérez‐Cembranos, León, & Pérez‐Mellado, 2016).

Although a trophic link with Raso larks was predictable, it was unexpected to find that this link is the strongest and is not restricted to the main breeding period of this lark (wet season). On the contrary, we found the highest percentage of positive records in July. Presently, the population of Raso lark has increased to more than 1,000 individuals due to favorable climate and it is known that this lark may breed during the dry season in some years (Ratcliffe, Monteiro, & Hazevoet, 1999). Whether the presence of this species in the fecal samples is the result of predation of viable eggs and nestlings, scavenging of nonviable eggs or dead fledged birds or ingestion of feces remains to be integrated with further information on seasonal mortality and the stochasticity of breeding events in the dry season. Considering all this, it is important to continue to monitor the impact of predation on this population, since its size can be severely reduced in drought years (Brooke, n.d.). In 2001, only 53 females were estimated, with a strong bias toward males (1.6 males per female) and the survival rate of nests was just 4.7%, mainly due to predation (Donald et al., 2003). At that time, the giant wall gecko was assumed to be the main or sole predator of Raso lark eggs, and since our data show that they are the most frequent vertebrate prey item found in its diet, this is highly probable.

The detection of DNA of Iago sparrow in gecko feces was also expected as the species is very abundant, present in most of the islet all year‐round, although no estimates of their population size are available. Geckos can easily access their eggs, since sparrows nest in rock crevices, which geckos also use as diurnal refuges. Iago sparrows are also threatened by the stochastic seasonal conditions that may promote adult and juvenile mortality and/or lower reproductive success in unfavorable years.

Seabirds have been considered one of the major items in the diet of this gecko, along with their regurgitations and feces that contain a large number of fish items. Observations clearly show that geckos also use cavities in rocks where seabirds nest or dwell. We found evidence for links with two of the most abundant species, the Cabo Verde shearwater and Bulwer's petrel. We did not find evidence of the other abundant seabird species, Cabo Verde storm petrel *Hydrobates jabejabe*. This may be explained due to their smaller size, for being more loosely colonial in comparison with the observed species, for using more burrows instead of rock crevices (this may occur in areas of lower gecko densities) and because some individuals breed in winter, factors that could decrease the opportunities for trophic interactions with the gecko during our sampling. The remaining seabird species either concentrate their nests in specific areas or their density is smaller. Overall, in recent years the number of breeding pairs of seabirds has been increasing steadily near the shoreline due to conservation measures, providing also more trophic resources. Although most seabirds only occur on Raso for breeding, each species has different breeding seasons, allowing the availability of these resources year‐round.

Our results concerning the occasional presence of fish in gecko feces need further integration with other sources of information to understand whether they are the result of the historic trophic ecology of seabird and/or raptors or due to anthropogenic influence. Indeed, all options are viable and they may not be mutually exclusive. Seabirds’ diet is mainly based on fish and cephalopods and geckos may be able to profit from the seabird regurgitations and solid feces, while the osprey *Pandion haliaetus* may leave discarded fish remains on the island. On the other hand, many fishes are handled and dried on the ground or eaten by fishermen that have their camps near the shoreline and that also perform their hygiene on land.

The confirmation of other potential but less relevant or more rare trophic links would require alternative or complementary approaches. The cannibalism of an juvenile specimen and the ingestion of a Cabo Verde wall gecko *Tarentola raziana* was found, at least, in one of the 50 feces samples that were morphologically analyzed previously (Mateo et al., 2016 ). However, the ingestion of the same species (cannibalism) and their feces (coprophagy) is not possible to discern using our protocol of DNA metabarcoding. Moreover, the blocking primer decreases the probability of amplifying DNA from *T. gigas* and *T. raziana*.

Ultimately, for some the vertebrate trophic links that were prioritized, the use of DNA metabarcoding was able to provide insights that would have been difficult to assess, but future integration with other techniques (e.g., focal observations or remote surveillance of passerines and seabirds breeding areas) could clarify the pathways and the type of relation (predation, scavenging, commensalism, mutualism) that occur with each species, while an assessment of this gecko foraging ranges could be helpful to understand the spatial impact of each gecko. However, these issues are out of the scope of this paper, since we did not focus on assessment of the whole diet of this gecko, but on the detection of trophic links with threatened vertebrates.

Considering the long‐term stability and viability of trophic links between this gecko and vertebrates, we showed that they rely mainly on the population dynamics of passerine and seabird species. Raso lark population dynamics is mainly correlated with annual rainfall (Brooke, n.d.; Brooke et al., 2012) and this is also probably the case for the Iago sparrow and the giant wall gecko populations. In a scenario of dry years, the predation pressure on passerine populations may be quite high, as already observed, and our data now corroborate that the giant wall gecko can be the main responsible for this pressure. In the case of the Raso lark, this raises conservation issues, due to the fact of being the only viable population of this species, while the Iago sparrow is widespread on the other islands of Cabo Verde. Seabirds were historically abundant but their populations declined due to human exploitation (Hazevoet, 2015). Only recently, after the creation of the Santa Luzia Nature Reserve, their populations increased. Our expectation is that the trophic link between geckos and birds may increase in the future and, in the best‐case scenario would only intensify the consumption of inviable young or eggs, regurgitations, and solid feces without impacting the fitness of adults. In this case, the link would be considered as a commensal or, if geckos act as phytosanitary agents, as a mutualistic relation. In the worst‐case scenario, this may have a negative impact on seabird egg and fledgling's survival due to predation. In this case, geckos would act as a major force of natural selection, driving species to extinction or promoting further adaptations to this harsh environment.

Our results are also informative to evaluate the viability of the reintroduction of the giant wall gecko on Santa Luzia and the impact on the ongoing reintroduction of Raso larks. Given that the frequency of occurrence of Raso larks in giant wall gecko diet was higher than for any other species detected, and that this gecko is suspected of predating Raso lark eggs (Donald et al., 2005,2003) we recommend that either gecko translocations are postponed until the Raso lark population is well‐established, or that geckos are translocated to a geographically separate portion of Santa Luzia Island, so as to minimize potential disturbance to Raso larks. Enclosures have been successfully used with the jeweled gecko (*Naultinus gemmeus*) for habituating animals to the release site, in order to restrict dispersal (Knox & Monks, 2014). In addition, the new population of Raso lark is rather small and they already face new invasive mammal predators that were not present on Raso, although measures are being taken to control them (Geraldes et al., 2016). On the other hand, Santa Luzia is a larger island, with higher habitat diversity and interactions can be minimized if their population sizes do not increase exponentially. But, as already stated before (Pinho et al., 2018), it is probably wise to model the impact of another predator on the viability and growth of this new Raso lark population, before any action is taken.

In conclusion, our results are an informative step toward understanding the ecological links between vertebrate species in this small system and how this ecological network is regulated. This final goal will require a clear knowledge about the functional trophic groups, and the importance of influxes of nutrients from the environment, seabirds, and human activities up to vertebrates, ultimately enhancing their integrated conservation.

## ETHICAL APPROVAL

Fieldwork with giant wall gecko was performed under permits from the DNA (Cabo Verde Direcção Nacional do Ambiente).

## CONFLICT OF INTEREST

None declared.

## AUTHORS' CONTRIBUTIONS

R.J.L. and R.V. designed and planned the study. C.J. P., B.S., and M.S. carried out the molecular laboratory work and analysis of reads and R.J.L., R.V, V.A.M., and B.E. analyzed and interpreted the data. R.J.L. drafted the manuscript with improvements from all authors. R.V. conceptualized the idea and coordinated the research activities. All authors read, commented, and approved the final manuscript.

## Data Availability

Data is available in the Dryad Digital Repository: https://doi.org/10.5061/dryad.sp10dk0.
